# Supplementary material for: Causes of death among people living with metastatic cancer
Source: Nat Commun. 2024 Feb 19;15:1519. doi: 10.1038/s41467-024-45307-x (PMC10876661; doi:10.1038/s41467-024-45307-x)
Supplement: Supplementary file 1 — Supplementary Information [file 41467_2024_45307_MOESM1_ESM.pdf]

SUPPLEMENTARY METHODS

Calendar period, registry, and diversity

The SEER program has evolved since its inception in the United States in 1973.[1] As of 2017, SEER has up to 36 years of longitudinal and ongoing data collection, with a representative sample size of more than 6 million cancer cases, and a comprehensive quality assurance process. Over time, more registries were added to SEER; in the current analysis, the SEER 18 (adjusted for Hurricane Katrina Impacted Louisiana cases) and SEER 9 registries were used. The registry number denotes the number of registries. *SEER 9.* The first areas included at that time were Connecticut, Hawaii, Iowa, San Francisco/Oakland, and Detroit. Geographic areas were included based on two objectives: (1) the ability of a geographic cancer registry to maintain high-quality data (explained below), and (2) having a population that represents minority subpopulations.[1]

In 1974-1975, the metropolitan areas of Atlanta and Seattle/Puget Sound were added, and the SEER 9 registry was finalized. *SEER 11.* In order to expand on the second objective, two more registries were added, Los Angeles County and 4 Counties in the San Jose/Monterey area. These counties included cases diagnosed after 1992. *SEER 13.* The next grouping additionally included 10 predominantly African American counties of rural Georgia and the Alaska Native American Tumor Registry. *SEER 17.* For cancers diagnoses after 2001, four additional areas were included: the remaining counties of California, Kentucky, Louisiana, and New Jersey. These counties have supplemental funding by the Centers for Disease Control (CDC). Based on the inclusion of these areas, the SEER database is representative of the population of the USA, and this has been validated by external studies.[1]

Since the SEER database have increased the proportion of the US population captured over the years, in early years of the SEER program there are fewer survivors than in later years, and the proportion of death by index cancer is lower in later years. Further, the rate count of people having a cancer depends on the number of patients living with this cancer from previous years (which depends on cancer prevalence), those diagnosed within the calendar year (which depends on screening and incidence), and those dying during that year (which depends on cancer and treatment aggressiveness, how death is coded, common risk factors among cancers and comorbidities, and patient age). Certain cancers have an indolent course (e.g. prostate), and patients diagnosed in subsequent years are added to the cumulative count, increasing the number of prostate cancer patients relative to all others; for patients with aggressive cancers (e.g. pancreatic), the addition of patients diagnosed in subsequent years has little effect on the cumulative number because of high rates of mortality.

Age

SEER provides age-standard adult (age ≥15) cancer populations to calculate age-standardized survival, which is used to compare survival across time or different cancer populations with different age distributions. The standards provided are the International Cancer Survival Standard (ICSS) derived for three broad groups of cancer sites with similar patterns of incidence by age. ICSS 1 includes cancer sites with increasing incidence by age (most cancer sites; e.g. prostate). ICSS 2 includes cancer sites with broadly constant incidence by age (e.g. nasopharynx). ICSS 3 includes cancer sites that mainly affect young adults (e.g. testis). By using the appropriate standard, the age-standardized survival is theorized to be like the raw (un-weighted) survival. For each of the three ICSS populations, SEER*Stat provides weights by 5-year age bins using the age variable, Age recode with <1 year olds, and by five larger age

groups, in the variable, Age Standard for Survival (15-44, 45-54, 55-64, 65-74, 75+), as described on the SEER website.

Quality assurance and completeness

SEER undergoes quality assurance using systematic, standardized, and periodic data collection procedure for all defined members of a defined cohort is performed to avoid surveillance bias.[1] The case-finding audits are performed by a qualified member from each SEER registry under the direction of members of the National Cancer Institute. Auditors create an abstract the contains the primary site and the case finding source.[2] When performing audits, SEER adheres to two basic principles: auditing high quantity and high risk data. High quantity refers to disease sites that have the highest incidence and prevalence (e.g. breast, prostate, lung, colon); as well facilities that contribute the greatest percent of cases to the central database. Additionally, pathology laboratories are selected to review tissue from patients not seen at that hospital. High risk refers to cases that are likely to be miscoded (e.g. head and neck, hematopoietic diseases); compliance to new rules; and newly-reportable diseases.

Defining the cause of death

Mortality codes in SEER are assigned from death certificates, completed by the doctor caring for the patient at the time of demise. There is no single best method for calculating survival from cancer in the SEER program.[3] Different methods can give different outcomes, but for most variants considered the differences are small.[3] For the purposes of the current work, for Objective I, data were characterized by rate count (i.e. the number of patients dying within a given year of all patients diagnosed, since 1992, with that particular cancer) and percentages of all deaths within a year, per 100,000 cases. The principal benefit of this method is that it allows us to include all patients alive in subsequent years after being diagnosed with a particular cancer, and calculate

their rate of death, relative to those with other cancers.

We do not present cause-specific survival because we were instead interested in finding a cause of death affecting a majority or plurality of patients with a particular diagnosis; thereby allowing us to identify patients who (1) are at lowest risk to die of their cancer; (2) are at highest risk to die of their cancer; (3) might profit from intense screening for second cancers; and (4) are at highest risk of non-cancer death (and its causes).

In early years of the SEER program there are fewer survivors than in later years, and the proportion of death by index cancer is lower in later years. Thus, per **Supplemental Figure 1**, the "rate count" of people having a cancer depends on the number of patients living with this cancer from previous years (which depends on cancer prevalence), those diagnosed within the calendar year (which depends on screening and incidence), and those dying during that year (which depends on cancer and treatment aggressiveness, how death is coded, common risk factors among cancers and comorbidities, and patient age). Certain cancers have an indolent course (e.g. prostate), and patients diagnosed in subsequent years are added to the cumulative count, increasing the number of prostate cancer patients relative to all others; for patients with aggressive cancers (e.g. pancreatic), the addition of patients diagnosed in subsequent years has little effect on the cumulative number because of high rates of mortality.

Cause of death came from patient death certificates. Death was coded as being as due to "deaths due to diagnosed cancer," (i.e. the cancer originally diagnosed in the patient), "deaths from subsequent-metastatic cancer" (i.e. a second site of metastases), and "deaths from non-cancer cause" (i.e. death from any medical cause not coded as cancer). Non-melanoma skin cancer deaths are not collected by the SEER registry and are excluded.

We examined multiple non-cancer causes of death, based on data from the Centers of Disease Control (CDC). Mortality codes in SEER are assigned from death certificates, completed by the doctor caring for the patient at the time of demise. Some fatalities attributed to a non-cancer cause may be due to early treatment complications (e.g. infection from chemotherapy for a hematologic malignancy). Hence, it is not possible to distinguish those deaths. For purposes of this analysis, unless "cancer" was the cause of death, it was considered referable to a medical cause. The latency period excludes synchronous primaries.

Data session information

The instructions to access the SEER data are provided below:

(1) Download the SEER*Stat software from the NCI website: https://seer.cancer.gov/seerstat/software/

(2) Open the program

(3) Click "File", "New,"

"MP-SIR" Session to generate the SMRs.

"Case Listing" to generate a list of patient cases diagnosed and cause of death data.

"Incidence" to generate a list of the incidence of cancer or cause of death.

Intricacies of Surveillance, Epidemiology, and End Results (SEER) Databases

Registry Differences

The SEER databases have been evolving over the years, and this evolution is described in our Data Availability Statement and by previous work.[1,4-6] Briefly, SEER covers key demographic areas in the United States, and these areas/databases have slowly been added to SEER since the 1970s. The SEER 9 database includes 9 registries from 1973-2014; the SEER 18 database

contains 18 registries, including the most recent patients from 2000-2016. Notably, SEER 18 is not limited to this time period; rather, the “2000-2016” refers to when all databases are collecting the data. Prior databases and their patients (before 2000) are available in SEER 18. The SEER 21 database was released in 2019, including more geographic regions. As data are collected from more regions, the same concepts of patient inclusion over time apply. SEER is able to analyze data by different methods, using its “Sessions.” The time period of these data sessions depend on the SEER database chosen (SEER 9, SEER 18, SEER 21, etc.). The “Standardized Incidence Ratio (SIR) session” provides incidence of a particular event after diagnosis, as a function of follow up time or age at diagnosis. When the event of interest is death as a function of follow up time, the SIRs are actually standardized mortality ratios (SMRs), and they provide the relative risk of death from a particular cause vs. the general population.

A “case listing” session is another option in displaying the data. Case listing sessions provide patient-level data, with each patient in a row, and variables (e.g. age, sex, cancer type) in columns. Thus, case listing sessions may be used to calculate odds ratios and generate survival plots.

Calculating Standardized Mortality Ratios

SMRs consist of two measures: (observed number of events, during time at risk) / (expected number of events in the reference population, during time at risk). SMRs may be calculated as a function of different times at risk, including time after diagnosis (i.e. the latency period) or age at diagnosis. When SMRs are calculated as a function of time after diagnosis, they provide the relative risk of death from one particular cause vs. the reference population. The reference population changes depending on the population and the time period. Thus, SMRs should not be compared to one another, and they would be expected to vary over different tim

periods or with different patient populations. Further, calculated SMRs may differ when using different SEER databases because (1) the observed number of events of interest among cancer patients may change, and (2) the number of events of interest in the reference population (i.e. the United States) also changes over the years.

Latency Exclusion Periods in Standardized Mortality Ratios

For SMRs calculated as a function of follow up time, SMRs during each window of time (e.g. at 1 year after diagnosis, 1-5 years after diagnosis, etc.) depend on the time at risk. With longer time at risk and more observed events, the confidence intervals become smaller, and measurements are more accurate. With a short time at risk (e.g. the first few months after diagnosis), or very few events (e.g. suicide), or among a niche patient cohort, the confidence intervals can widen dramatically.

In the first few months after diagnosis of cancer, patients often have an "introduction to the medical system;" i.e. a patient living in a rural area comes to a hospital where they are diagnosed with cancer, as well as many other comorbidities like heart disease, lung dysfunction, kidney failure, etc. The patient may die of any of these within a few months, but estimating the observed versus expected rate of death becomes difficult, and the confidence intervals for an SMR naturally widen. Thus, some researchers, including our team, sometimes elect to exclude the first 2 months from the SMR calculations. While SMRs may actually be very high during this time, the confidence intervals are so wide that an accurate measure is not meaningful. Moreover, the absolute number of observed events in this time may be rather low, especially when the event of interest is rare. Thus, the overall SMRs for the entire follow up period (with or without the latency periods) tend to be relatively similar.

SUPPLEMENTAL TABLES.

Supplementary Table 1. Causes of noncancer death among patients living with metastatic cancer who were actively followed in 2020 and were at risk of fatal COVID-19 infection.

**Cause of death**	Non-cancer Death counts[A]	Survival (Months)[A]
Accidents and Adverse Effects	337 (4.20%)	31.0
Alzheimer's (ICD-9 and ICD-10 only)	235 (2.93%)	86.0
COVID (2020+ only)	1039 (12.96%)	36.0
Cerebrovascular diseases	441 (5.50%)	43.0
Chronic Liver Disease and Cirrhosis	102 (1.27%)	21.5
Chronic Obstructive Pulmonary Disease and Allied Conditions	437 (5.45%)	38.0
Complications of medical and surgical care (Y40-Y84, Y88) (ICD-10 only, 1999+)	38 (0.47%)	29.0
Congenital Anomalies	13 (0.16%)	26.0
Diabetes Mellitus	235 (2.93%)	52.0
Diseases of arteries, arterioles and capillaries	68 (0.85%)	46.5
Homicide and Legal Intervention	9 (0.11%)	21.0
Hypertensive disease	334 (4.17%)	46.0
Ischemic heart disease	1118 (13.95%)	42.5
Nephritis, Nephrotic Syndrome and Nephrosis	187 (2.33%)	48.0
Other COD	1595 (19.9%)	38.0
Other and unspecified disorders of the circulatory system	666 (8.31%)	40.0
Other infectious and Parasitic Diseases incl HIV	188 (2.35%)	15.0
Pneumonia and Influenza	280 (3.49%)	30.0
Pulmonary heart disease and diseases of pulmonary circulation	75 (0.94%)	28.0
Septicemia	217 (2.71%)	17.0
Soft Tissue of the Heart	228 (2.84%)	11.0
Stomach and Duodenal Ulcers	14 (0.17%)	60.0
Suicide and Self-Inflicted Injury	59 (0.74%)	14.0
Symptoms, Signs and Ill-Defined Conditions	100 (1.25%)	29.5

[A] Database “SEER Research Data, 12 Registries, Nov 2021 Sub (1992-2020) was used for death counts and survival months. The median survival time is defined as the length of time from the initial cancer diagnosis at which half of the patients are alive.

Supplemental Table 2. Clinical descriptions for the non-neoplasm causes of death using SEER's standard 1969+ Recode

Non-Neoplasm Causes of Death[A]	ICD-10 Code[A]	ICD-10 Code Clinical[B] Description
**Tuberculosis**	A15-A19	A15-A19 Tuberculosis
**Syphilis**	A50-A53	A50-A53 Syphilis
**Human Immunodeficiency Virus (HIV) (1987+)**	B20-B24	B20-B24 HIV
**Septicemia**	A40-A41	A40 Streptococcal sepsis<br>A41 Other sepsis
**Other Infectious and Parasitic Diseases**	A00-A08, A20-A33, A35-A39, A42-A49, A54-B19, B25-B99	E10 Type 1 diabetes mellitus<br>E11 Type 2 diabetes mellitus<br>E13 Other specified diabetes mellitus
**Diabetes Mellitus**	E10-E14	E10 Type 1 diabetes mellitus<br>E11 Type 2 diabetes mellitus<br>E13 Other specified diabetes mellitus
**Alzheimers (ICD-9 and 10 only)**	G30	Alzheimer's
**Diseases of Heart**	I00-I09, I11, I13, I20-I51	I00-I02 Acute rheumatic fever<br>I05-I09 Chronic rheumatic heart disease<br>I11 Hypertensive heart disease<br>I13 Hypertensive heart and chronic kidney disease<br>I20 Angina pectoris<br>I21 Acute myocardial infarction<br>I22 Subsequent ST elevation (STEMI) and non-ST elevation (NSTEMI) myocardial infarction<br>I23 Certain current complications following STEMI and NSTEMI myocardial infraction<br>I24 Other acute ischemic heart disease<br>I25 Chronic ischemic heart disease<br>I26 Pulmonary embolism<br>I27 Other pulmonary heart diseases<br>I28 Other diseases of pulmonary vessels<br>I30-I51 Other forms of heart disease
**Hypertension without Heart Disease**	I10, I12	I10 Essential (primary) hypertension<br>I12 Hypertensive chronic kidney disease
**Cerebrovascular Diseases**	I60-I69	I60 Nontraumatic subarachnoid hemorrhage<br>I61 Nontraumatic intracerebral hemorrhage<br>I62 Other and unspecified nontraumatic intracranial hemorrhage<br>I63 Cerebral infarction<br>I65 Occlusion and stenosis of precerebral arteries, not resulting in cerebral infarction<br>I66 Occlusion and stenosis of cerebral arteries, not resulting in cerebral infarction<br>I67 Other cerebrovascular diseases<br>I68 Cerebrovascular disorders in diseases classified elsewhere<br>I69 Sequelae of cerebrovascular disease
**Atherosclerosis**	I70	I70 Atherosclerosis
**Aortic Aneurysm and Dissection**	I71	I71 Aortic aneurysm and dissection
**Other Diseases of Arteries, Arterioles, Capillaries**	I72-I78	I72 Other aneurysm<br>I73 Other peripheral vascular diseases<br>I74 Arterial embolism and thrombosis<br>I75 Aeroembolism<br>I76 Septic arterial embolism<br>I77 Other disorders of arteries and arterioles<br>I78 Diseases of capillaries

Non-Neoplasm Causes of Death[A]	ICD-10 Code[A]	ICD-10 Code Clinical[B] Description
**Pneumonia and Influenza**	J09-J18	J09 Influenza due to certain identified influenza viruses<br>J10 Influenza due to other identified influenza virus<br>J11 Influenza due to unidentified influenza virus<br>J12 Viral pneumonia, not elsewhere classified<br>J13 Pneumonia due to Streptococcus pneumoniae<br>J14 Pneumonia due to Hemophilus influenzae<br>J15 Bacterial pneumonia, not elsewhere classified<br>J16 Pneumonia due to other infectious organisms, not elsewhere classified<br>J17 Pneumonia in diseases classified elsewhere<br>J18 Pneumonia, unspecified organism
**Chronic Obstructive Pulmonary Disease and Allied Cond**	J40-J47	J40 Bronchitis, not specified as acute or chronic<br>J41 Simple and mucopurulent chronic bronchitis<br>J42 Unspecified chronic bronchitis<br>J43 Emphysema<br>J44 Other chronic obstructive pulmonary disease<br>J45 Asthma<br>J47 Bronchiectasis
**Stomach and Duodenal Ulcers**	K25-K28	K25 Gastric ulcer<br>K26 Duodenal ulcer<br>K27 Peptic ulcer, site unspecified<br>K28 Gastrojejunal ulcer
**Chronic Liver Disease and Cirrhosis**	K70, K73-K74	K70 Alcoholic liver disease<br>K73 Chronic hepatitis, not elsewhere classified<br>K74 Fibrosis and cirrhosis of live
**Nephritis, Nephrotic Syndrome and Nephrosis**	N00-N07, N17-N19, N25-N27	N00-N08 Glomerular diseases<br>N17-N19 Acute kidney failure and chronic kidney disease<br>N25 Disorders resulting from impaired renal tubular function<br>N26 Unspecified contracted kidney<br>N27 Small kidney of unknown cause
**Complications of Pregnancy, Childbirth, Puerperium**	A34, O00-O95, O98-O99	A34 Obstetrical tetanus<br>O00-O08 Pregnancy with abortive outcome<br>O09-O09 Supervision of high risk pregnancy<br>O10-O16 Edema, proteinuria and hypertensive disorders in pregnancy, childbirth and the puerperium<br>O20-O29 Other maternal disorders predominantly related to pregnancy<br>O30-O48 Maternal care related to the fetus and amniotic cavity and possible delivery problems<br>O60-O77 Complications of labor and delivery<br>O80-O82 Encounter for delivery<br>O85-O92 Complications predominantly related to the puerperium<br>O94-O9A Other obstetric conditions, not elsewhere classified
**Congenital Anomalies**	Q00-Q99	Q00-Q07 Congenital malformations of the nervous system<br>Q10-Q18 Congenital malformations of eye, ear, face and neck<br>Q20-Q28 Congenital malformations of the circulatory system<br>Q30-Q34 Congenital malformations of the respiratory system<br>Q35-Q37 Cleft lip and cleft palate<br>Q38-Q45 Other congenital malformations of the digestive system<br>Q50-Q56 Congenital malformations of genital organs<br>Q60-Q64 Congenital malformations of the urinary system

Non-Neoplasm Causes of Death[A]	ICD-10 Code[A]	ICD-10 Code Clinical[B] Description
		Q65-Q79 Congenital malformations and deformations of the musculoskeletal system<br>Q80-Q89 Other congenital malformations<br>Q90-Q99 Chromosomal abnormalities, not elsewhere classified
**Certain Conditions Originating in Perinatal Period**	P00-P96	P00-P04 Newborn affected by maternal factors and by complications of pregnancy, labor, and delivery<br>P05-P08 Disorders of newborn related to length of gestation and fetal growth<br>P09-P09 Abnormal findings on neonatal screening<br>P10-P15 Birth trauma<br>P19-P29 Respiratory and cardiovascular disorders specific to the perinatal period<br>P35-P39 Infections specific to the perinatal period<br>P50-P61 Hemorrhagic and hematological disorders of newborn<br>P70-P74 Transitory endocrine and metabolic disorders specific to newborn<br>P76-P78 Digestive system disorders of newborn<br>P80-P83 Conditions involving the integument and temperature regulation of newborn<br>P84-P84 Other problems with newborn<br>P90-P96 Other disorders originating in the perinatal period
**Accidents and Adverse Effects**	V01-X59, Y85-Y86	V00-V09 Pedestrian injured in transport accident<br>V10-V19 Pedal cycle rider injured in transport accident<br>V20-V29 Motorcycle rider injured in transport accident<br>V30-V39 Occupant of three-wheeled motor vehicle injured in transport accident<br>V40-V49 Car occupant injured in transport accident<br>V50-V59 Occupant of pick-up truck or van injured in transport accident<br>Y85-Y89 Sequelae of external causes of morbidity and mortality
**Suicide and Self-Inflicted Injury**	U03, X60-X84, Y870	U03 Intentional self-harm (suicide)<br>X60-X84, Y870 Self-inflicted injuries
**Symptoms, Signs and Ill-Defined Conditions**	R00-R99	R00-R09 Symptoms and signs involving the circulatory and respiratory systems<br>R10-R19 Symptoms and signs involving the digestive system and abdomen<br>R20-R23 Symptoms and signs involving the skin and subcutaneous tissue<br>R25-R29 Symptoms and signs involving the nervous and musculoskeletal systems<br>R30-R39 Symptoms and signs involving the genitourinary system<br>R40-R46 Symptoms and signs involving cognition, perception, emotional state and behavior<br>R47-R49 Symptoms and signs involving speech and voice<br>R50-R69 General symptoms and signs<br>R70-R79 Abnormal findings on examination of blood, without diagnosis

Non-Neoplasm Causes of Death[A]	ICD-10 Code[A]	ICD-10 Code Clinical[B] Description
		R80-R82 Abnormal findings on examination of urine, without diagnosis<br>R83-R89 Abnormal findings on examination of other body fluids, substances and tissues, without diagnosis<br>R90-R94 Abnormal findings on diagnostic imaging and in function studies, without diagnosis<br>R97-R97 Abnormal tumor markers<br>R99-R99 Ill-defined and unknown cause of mortality
**Homicide and Legal Intervention**	U01-U02, X85-Y09, Y35, Y871, Y890	U01-U02, X85-Y09, 87 Homicide - All injury<br>Y35 Legal Intervention/War - All injury

[A]Non-Neoplasm Causes of Death and ICD-0-10 columns adapted from SEER: https://seer.cancer.gov/codrecode/
[B]ICD-0-10 clinical description column adapted from the World Health Organization: https://icd.who.int/ and https://www.icd10data.com/

Supplementary Table 3. Causes of noncancer death among patients living with metastatic cancer using the alternative ICD-10 2023+ Revision, 1992-2019

**Cause of death**	Non-cancer Death counts[A]	Survival (Months)[A]
Accidents and Adverse Effects	4091 (3.55%)	25
Alzheimer's (ICD-9 and ICD-10 only)	2704 (2.35%)	66
Cerebrovascular diseases	7126 (6.18%)	20
Certain Conditions Originating in Perinatal Period	19 (0.02%)	1
Chronic Liver Disease and Cirrhosis	1689 (1.47%)	9
Chronic Obstructive Pulmonary Disease and Allied Conditions	9194 (7.98%)	15
Complications of medical and surgical care (Y40-Y84, Y88) (ICD-10 only, 1999+)	349 (0.30%)	18
Congenital Anomalies	260 (0.23%)	9
Diabetes Mellitus	3354 (2.91%)	22
Diseases of arteries, arterioles and capillaries	1759 (1.53%)	15
Homicide and Legal Intervention	91 (0.08%)	23
Hypertensive disease	3681 (3.19%)	26
Ischemic heart disease	23331 (20.24%)	16
Nephritis, Nephrotic Syndrome and Nephrosis	2897 (2.51%)	25
Other COD	21217 (18.41%)	20
Other and unspecified disorders of the circulatory system	11541 (10.01%)	21
Other infectious and Parasitic Diseases incl HIV	4849 (4.21%)	7
Pneumonia and Influenza	5359 (4.65%)	15
Pulmonary heart disease and diseases of pulmonary circulation	1087 (0.94%)	15
Septicemia	2765 (2.40%)	12
Soft Tissue	4403 (3.82%)	9
Stomach and Duodenal Ulcers	451 (0.39%)	8
Suicide and Self-Inflicted Injury	1233 (1.07%)	10

[A] Database "SEER Research Data, 12 Registries, Nov 2021 Sub (1992-2020) was used for death counts and survival months. The median survival time is defined as the length of time from the initial cancer diagnosis at which half of the patients are alive.

Supplementary Table 4. Clinical descriptions for the non-neoplasm causes of death using SEER's alternate 2023+ Recode.

Non-Neoplasm Causes of Death[A]	ICD-10 Code[A]	ICD-10 Code Clinical[B] Description
**HIV**	B20-B24	HIV
**Septicemia**	A40-A41	A40 Streptococcal sepsis<br>A41 Other sepsis
**Other infectious and Parasitic Diseases**	A00-A08, A15-A33, A35-A39, A42-B19, B25-B99)	A00-A09 Intestinal infectious diseases<br>A15-A19 Tuberculosis<br>A20-A28 Certain zoonotic bacterial diseases<br>A30-A49 Other bacterial diseases<br>A50-A64 Infections with a predominantly sexual mode of transmission<br>A65-A69 Other spirochetal diseases<br>A70-A74 Other diseases caused by chlamydia<br>A75-A79 Rickettsioses<br>A80-A89 Viral and prion infections of the central nervous system<br>A90-A99 Arthropod-borne viral fevers and viral hemorrhagic fevers<br>B00-B09 Viral infections characterized by skin and mucous membrane lesions<br>B10-B10 Other human herpesviruses<br>B15-B19 Viral hepatitis<br>B25-B34 Other viral diseases<br>B35-B49 Mycoses<br>B50-B64 Protozoal diseases<br>B65-B83 Helminthiases<br>B85-B89 Pediculosis, acariasis and other infestations<br>B90-B94 Sequelae of infectious and parasitic diseases<br>B95-B97 Bacterial and viral infectious agents<br>B99-B99 Other infectious diseases
**COVID (2020+ only)**	U071	COVID-19
**Diabetes Mellitus**	E10-E14	E10 Type 1 diabetes mellitus<br>E11 Type 2 diabetes mellitus<br>E13 Other specified diabetes mellitus
**Alzheimer's (ICD-9 and ICD-10 only)**	G30	Alzheimer's
**Circulatory Disease**		
**Hypertensive disease**	I10-I15	I10 Essential (primary) hypertension<br>I11 Hypertensive heart disease<br>I12 Hypertensive chronic kidney disease<br>I13 Hypertensive heart and chronic kidney disease<br>I15 Secondary hypertension<br>I16 Hypertensive crisis
**Ischemic heart disease**	I20-I25	I20 Angina pectoris<br>I21 Acute myocardial infarction<br>I22 Subsequent ST elevation (STEMI) and non-ST elevation (NSTEMI) myocardial infarction<br>I23 Certain current complications following STEMI and NSTEMI myocardial infraction<br>I24 Other acute ischemic heart disease<br>I25 Chronic ischemic heart disease
**Pulmonary heart disease and diseases of pulmonary circulation**	I26-I28	I26 Pulmonary embolism<br>I27 Other pulmonary heart diseases<br>I28 Other diseases of pulmonary vessels

Non-Neoplasm Causes of Death[A]	ICD-10 Code[A]	ICD-10 Code Clinical[B] Description
**Cerebrovascular diseases**	I60-I69	I60 Nontraumatic subarachnoid hemorrhage<br>I61 Nontraumatic intracerebral hemorrhage<br>I62 Other and unspecified nontraumatic intracranial hemorrhage<br>I63 Cerebral infarction<br>I65 Occlusion and stenosis of precerebral arteries, not resulting in cerebral infarction<br>I66 Occlusion and stenosis of cerebral arteries, not resulting in cerebral infarction<br>I67 Other cerebrovascular diseases<br>I68 Cerebrovascular disorders in diseases classified elsewhere<br>I69 Sequelae of cerebrovascular disease
**Diseases of arteries, arterioles, and capillaries**	I70-I78	I70 Atherosclerosis<br>I71 Aortic aneurysm and dissection<br>I72 Other aneurysm<br>I73 Other peripheral vascular diseases<br>I74 Arterial embolism and thrombosis<br>I75 Aeroembolism<br>I76 Septic arterial embolism<br>I77 Other disorders of arteries and arterioles<br>I78 Diseases of capillaries
**Other and unspecified disorders of the circulatory system**	I00-I09, I30-I51, I80-I99	I00-I02 Acute rheumatic fever<br>I05-I09 Chronic rheumatic heart diseases<br>I30-I51 Other forms of heart disease<br>I80-I89 Diseases of veins, lymphatic vessels and lymph nodes, not elsewhere classified
**Pneumonia and Influenza**	J09-J18	J09 Influenza due to certain identified influenza viruses<br>J10 Influenza due to other identified influenza virus<br>J11 Influenza due to unidentified influenza virus<br>J12 Viral pneumonia, not elsewhere classified<br>J13 Pneumonia due to Streptococcus pneumoniae<br>J14 Pneumonia due to Hemophilus influenzae<br>J15 Bacterial pneumonia, not elsewhere classified<br>J16 Pneumonia due to other infectious organisms, not elsewhere classified<br>J17 Pneumonia in diseases classified elsewhere<br>J18 Pneumonia, unspecified organism
**Chronic Obstructive Pulmonary Disease and Allied Conditions**	J40-J47	J40 Bronchitis, not specified as acute or chronic<br>J41 Simple and mucopurulent chronic bronchitis<br>J42 Unspecified chronic bronchitis<br>J43 Emphysema<br>J44 Other chronic obstructive pulmonary disease<br>J45 Asthma<br>J47 Bronchiectasis
**Stomach and Duodenal Ulcers**	K25-K28	K25 Gastric ulcer<br>K26 Duodenal ulcer<br>K27 Peptic ulcer, site unspecified<br>K28 Gastrojejunal ulcer
**Chronic Liver Disease and Cirrhosis**	K70, K73-K74	K70 Alcoholic liver disease<br>K73 Chronic hepatitis, not elsewhere classified<br>K74 Fibrosis and cirrhosis of live
**Nephritis, Nephrotic Syndrome and Nephrosis**	N00-N07, N17-N19, N25-N27	N00-N08 Glomerular diseases<br>N17-N19 Acute kidney failure and chronic kidney disease<br>N25 Disorders resulting from impaired renal tubular function<br>N26 Unspecified contracted kidney<br>N27 Small kidney of unknown cause

Non-Neoplasm Causes of Death[A]	ICD-10 Code[A]	ICD-10 Code Clinical[B] Description
**Complications of Pregnancy, Childbrith, Puerperium**	A34, O00-O95, O98-O99	A34 Obstetrical tetanus<br>O00-O08 Pregnancy with abortive outcome<br>O09-O09 Supervision of high risk pregnancy<br>O10-O16 Edema, proteinuria and hypertensive disorders in pregnancy, childbirth and the puerperium<br>O20-O29 Other maternal disorders predominantly related to pregnancy<br>O30-O48 Maternal care related to the fetus and amniotic cavity and possible delivery problems<br>O60-O77 Complications of labor and delivery<br>O80-O82 Encounter for delivery<br>O85-O92 Complications predominantly related to the puerperium<br>O94-O9A Other obstetric conditions, not elsewhere classified
**Congenital Anomalies**	Q00-Q99	Q00-Q07 Congenital malformations of the nervous system<br>Q10-Q18 Congenital malformations of eye, ear, face and neck<br>Q20-Q28 Congenital malformations of the circulatory system<br>Q30-Q34 Congenital malformations of the respiratory system<br>Q35-Q37 Cleft lip and cleft palate<br>Q38-Q45 Other congenital malformations of the digestive system<br>Q50-Q56 Congenital malformations of genital organs<br>Q60-Q64 Congenital malformations of the urinary system<br>Q65-Q79 Congenital malformations and deformations of the musculoskeletal system<br>Q80-Q89 Other congenital malformations<br>Q90-Q99 Chromosomal abnormalities, not elsewhere classified
**Certain Conditions Originating in Perinatal Period**	P00-P96	P00-P04 Newborn affected by maternal factors and by complications of pregnancy, labor, and delivery<br>P05-P08 Disorders of newborn related to length of gestation and fetal growth<br>P09-P09 Abnormal findings on neonatal screening<br>P10-P15 Birth trauma<br>P19-P29 Respiratory and cardiovascular disorders specific to the perinatal period<br>P35-P39 Infections specific to the perinatal period<br>P50-P61 Hemorrhagic and hematological disorders of newborn<br>P70-P74 Transitory endocrine and metabolic disorders specific to newborn<br>P76-P78 Digestive system disorders of newborn<br>P80-P83 Conditions involving the integument and temperature regulation of newborn<br>P84-P84 Other problems with newborn<br>P90-P96 Other disorders originating in the perinatal period

Non-Neoplasm Causes of Death[A]	ICD-10 Code[A]	ICD-10 Code Clinical[B] Description
**Symptoms, Signs and Ill-Defined Conditions**	R00-R99	R00-R09 Symptoms and signs involving the circulatory and respiratory systems<br>R10-R19 Symptoms and signs involving the digestive system and abdomen<br>R20-R23 Symptoms and signs involving the skin and subcutaneous tissue<br>R25-R29 Symptoms and signs involving the nervous and musculoskeletal systems<br>R30-R39 Symptoms and signs involving the genitourinary system<br>R40-R46 Symptoms and signs involving cognition, perception, emotional state and behavior<br>R47-R49 Symptoms and signs involving speech and voice<br>R50-R69 General symptoms and signs<br>R70-R79 Abnormal findings on examination of blood, without diagnosis<br>R80-R82 Abnormal findings on examination of urine, without diagnosis<br>R83-R89 Abnormal findings on examination of other body fluids, substances and tissues, without diagnosis<br>R90-R94 Abnormal findings on diagnostic imaging and in function studies, without diagnosis<br>R97-R97 Abnormal tumor markers<br>R99-R99 Ill-defined and unknown cause of mortality
**Accidents and Adverse Effects**	V01-X59, Y85-Y86	V00-V09 Pedestrian injured in transport accident<br>V10-V19 Pedal cycle rider injured in transport accident<br>V20-V29 Motorcycle rider injured in transport accident<br>V30-V39 Occupant of three-wheeled motor vehicle injured in transport accident<br>V40-V49 Car occupant injured in transport accident<br>V50-V59 Occupant of pick-up truck or van injured in transport accident<br>Y85-Y89 Sequelae of external causes of morbidity and mortality
**Suicide and Self-Inflicted Injury**	U03, X60-X84, Y870	U03 Intentional self-harm (suicide)<br>X60-X84, Y870 Self-inflicted injuries
**Homicide and Legal Intervention**	U01-U02, X85-Y09, Y35, Y871, Y890	U01-U02, X85-Y09, 87 Homicide - All injury<br>Y35 Legal Intervention/War - All injury
**Complications of medical and surgical care (ICD-10 only, 1999+)**	Y40-Y84, Y88	Y40-Y59 Drugs, medicaments and biological substances causing adverse effects in therapeutic use<br>Y60-Y69 Misadventures to patients during surgical and medical care<br>Y70-Y82 Medical devices associated with adverse incidents in diagnostic and therapeutic use<br>Y83-Y84 Surgical and other medical procedures as the cause of abnormal reaction of the patient, or of later complication, without mention of misadventure at the time of the procedure<br>Y88:Sequelae with surgical and medical care as external cause

[A]Non-Neoplasm Causes of Death and ICD-0-10 columns adapted from SEER: https://seer.cancer.gov/codrecode/
[B]ICD-0-10 clinical description column adapted from the World Health Organization: https://icd.who.int/ and https://www.icd10data.com/

Supplemental Table 5. Sub-distribution Hazard ratios (SHR) and β coefficients for the Fine-Gray competing risk models.

	Death due to diagnosed cancer		Death due to non-cancer causes	
	SHR (95% CI)	*p*-value[a]	SHR (95% CI)	*p*-value[a]
Age Group				
<=54	1.00	--	1.00	--
55-64	1.17 (1.15-1.18)	<0.001	1.51 (1.42, 1.61)	<0.001
65-74	1.25 (1.24-1.27)	<0.001	2.06 (1.94, 2.19)	<0.001
75-84	1.50 (1.47-1.52)	<0.001	2.71 (2.55, 2.88)	<0.001
85+	1.89 (1.85-1.93)	<0.001	3.46 (3.22, 3.69)	<0.001
Sex				
Male	1.00	--	1.00	--
Female	0.94 (0.93-0.95)	<0.001	0.87 (0.84, 0.90)	<0.001
Race				
White	1.00	--	1.00	--
Black	1.06 (1.04-1.08)	<0.001	1.32 (1.27, 1.38)	<0.001
AI/AN	1.05 (1.00-1.10)	0.041	1.43 (1.27, 1.61)	<0.001
API	0.89 (0.88-0.90)	<0.001	0.86 (0.82, 0.90)	<0.001
T-stage				
TX	1.00	--	1.00	--
T1	0.70 (0.68-0.71)	<0.001	1.00 (0.95, 1.06)	0.93
T2	0.79 (0.77, 0.80)	<0.001	0.91 (0.87, 0.95)	<0.001
T3	0.77 (0.76, 0.78)	<0.001	0.86 (0.82, 0.90)	<0.001
T4	0.83 (0.82, 0.85)	<0.001	0.88 (0.84, 0.92)	<0.001
N-stage				
NX	1.00	--	1.00	--
N1	1.02 (1.01, 1.03)	0.005	0.83 (0.80, 0.87)	<0.001
N2	1.14 (1.12, 1.15)	<0.001	0.76 (0.73, 0.79)	<0.001
N3	1.08 (1.06, 1.10)	<0.001	0.78 (0.74, 0.82)	<0.001
Bone Mets				
Yes	1.00	--	1.00	--
No	1.29 (1.27, 1.30)	<0.001	0.78 (0.75, 0.81)	<0.001
Brain Mets				
Yes	1.00	--	1.00	--
No	1.33 (1.31, 1.35)	<0.001	0.68 (0.65, 0.73)	<0.001
Liver Mets				
Yes	1.00	--	1.00	--
No	1.42 (1.41, 1.44)	<0.001	0.78 (0.75, 0.81)	<0.001
Lung Mets				
Yes	1.00	--	1.00	--
No	1.18 (1.17, 1.20)	<0.001	0.92 (0.89, 0.96)	<0.001
Primary Cancer Site				

Breast	1.00	--	1.00	--
Lung and Bronchus	2.12 (2.07, 2.16)	<0.001	0.98 (0.92, 1.05)	0.60
Ovary	1.41 (1.37, 1.45)	<0.001	0.59 (0.53, 0.65)	<0.001
Urinary Bladder	2.14 (2.05, 2.24)	<0.001	0.98 (0.87, 1.11)	0.77
Pancreas	3.14 (3.06, 3.23)	<0.001	0.51 (0.47, 0.56)	<0.001
Esophagus	2.42 (2.34, 2.51)	<0.001	0.72 (0.64, 0.82)	<0.001
Prostate	0.75 (0.73, 0.78)	<0.001	1.16 (1.07, 1.26)	<0.001
Kidney and Renal Pelvis	1.80 (1.74, 1.86)	<0.001	0.87 (0.79, 0.96)	<0.001
Colon	1.56 (1.52, 1.61)	<0.001	0.78 (0.72, 0.85)	<0.001
Oral Cavity and Pharynx	1.02 (0.98, 1.06)	0.420	1.45 (1.32, 1.60)	<0.001
Melanoma of the Skin	1.05 (1.00, 1.10)	0.061	1.11 (0.98, 1.26)	0.10
Stomach	2.50 (2.43, 2.58)	<0.001	0.62 (0.56, 0.69)	<0.001
Rectum	1.40 (1.36, 1.44)	<0.001	0.71 (0.64, 0.80)	<0.001
Liver and Bile Duct	3.53 (3.40, 3.68)	<0.001	0.71 (0.63, 0.80)	<0.001
Corpus Uteri	1.63 (1.57, 1.69)	<0.001	0.74 (0.66, 0.84)	<0.001

a: The Wald test was performed. Type III. P-values are two-sided. No adjustment for multiple comparisons were made.

SUPPLEMENTARY FIGURE 1

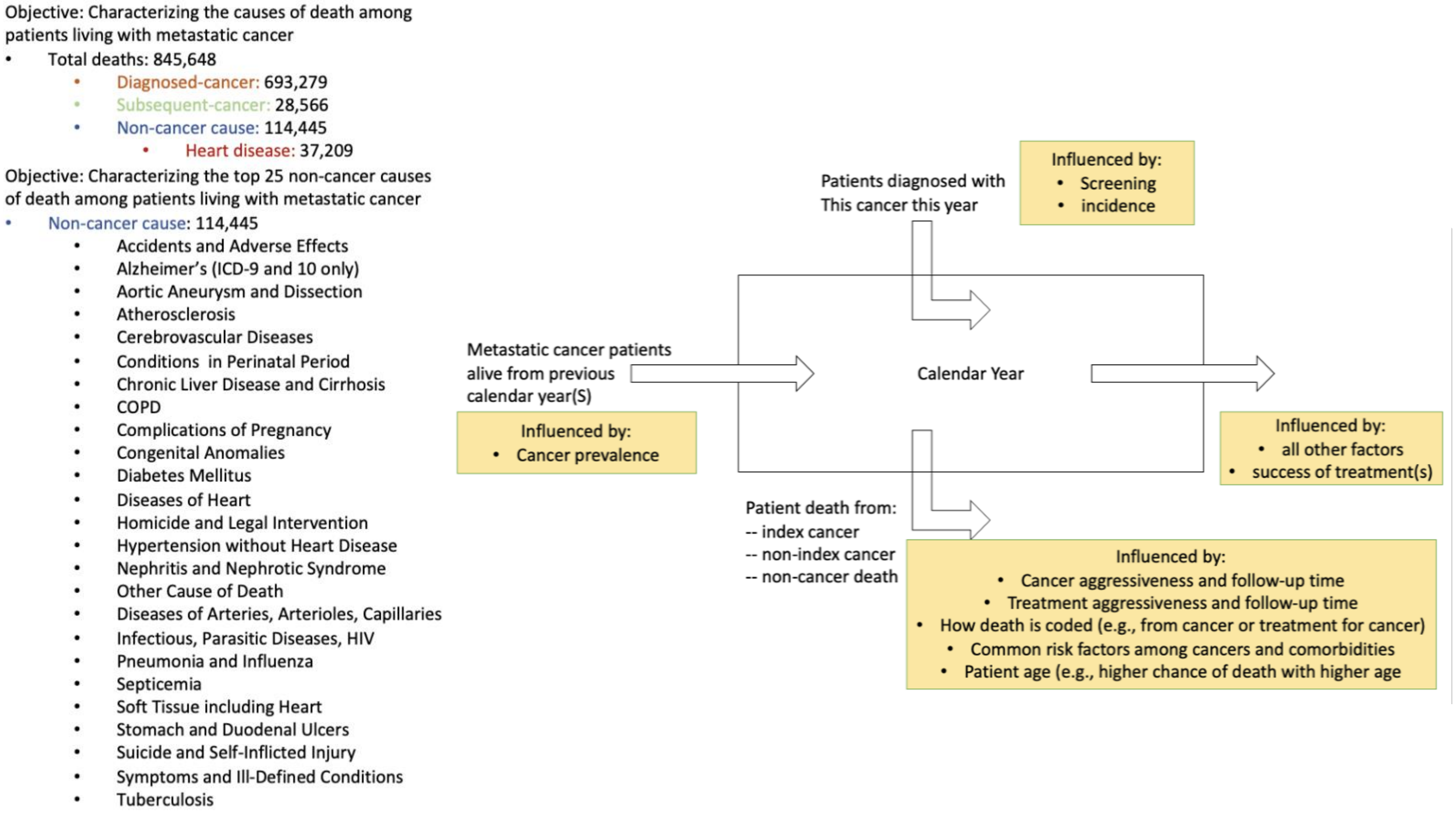

Supplemental Figure 1. Patient numbers for methodology objectives. Orange color indicates diagnosed cancer patient numbers, green color indicates subsequent cancer patient numbers, blue color indicates non-cancer cause of death patient numbers, and red color indicates heart disease mortality patient numbers. Objective I: calculation of death rates per year. The calculation of mortality per calendar year is influenced by several factors, such as cancer prevalence, screening, incidence, success of treatments, both cancer and treatment aggressiveness and follow-up times, patient age, how death is coded, and common risk factors among cancer patients, including comorbidities.

SUPPLEMENTARY FIGURE 2

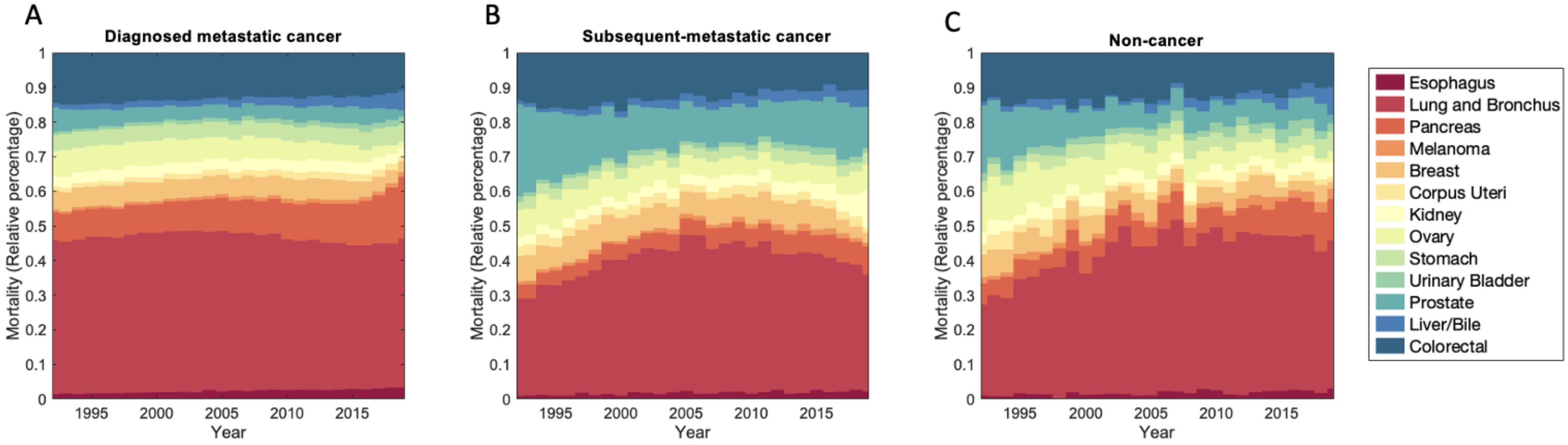

Supplemental Figure 2. Plots of the proportion of all metastatic cancer patients that experience death due to A) primary cancer only, B) secondary cancer only, and C) all other medical causes of death only, stratified by individual cancer subtypes.

SUPPLEMENTARY FIGURE 3

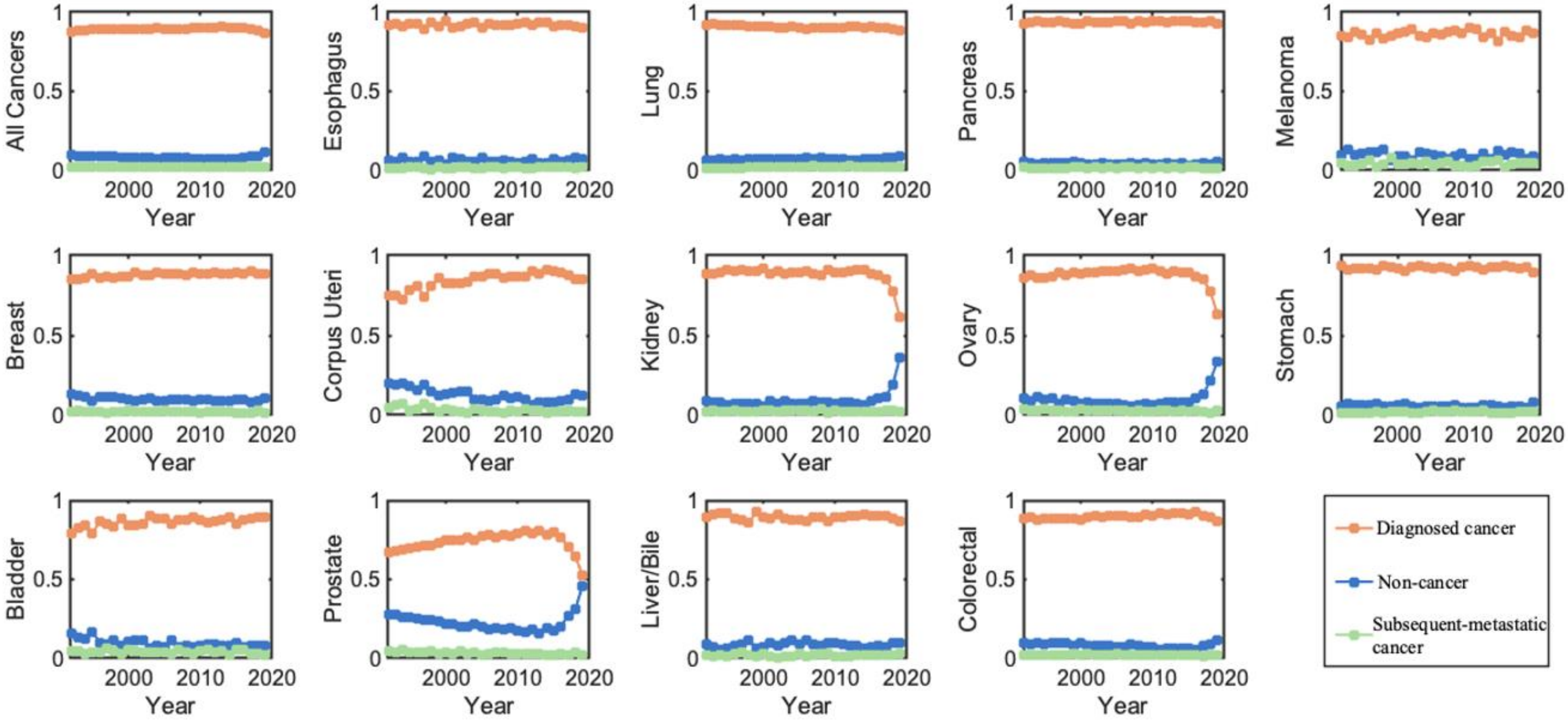

Supplemental Figure 3. Plots of relative mortality counts versus year of diagnosis (1992-2019) for various metastatic cancer subtypes. Death was stratified due to primary cancer (the cancer originally diagnosed by the patient), secondary cancer, or all other medical causes of death.

SUPPLEMENTARY FIGURE 4

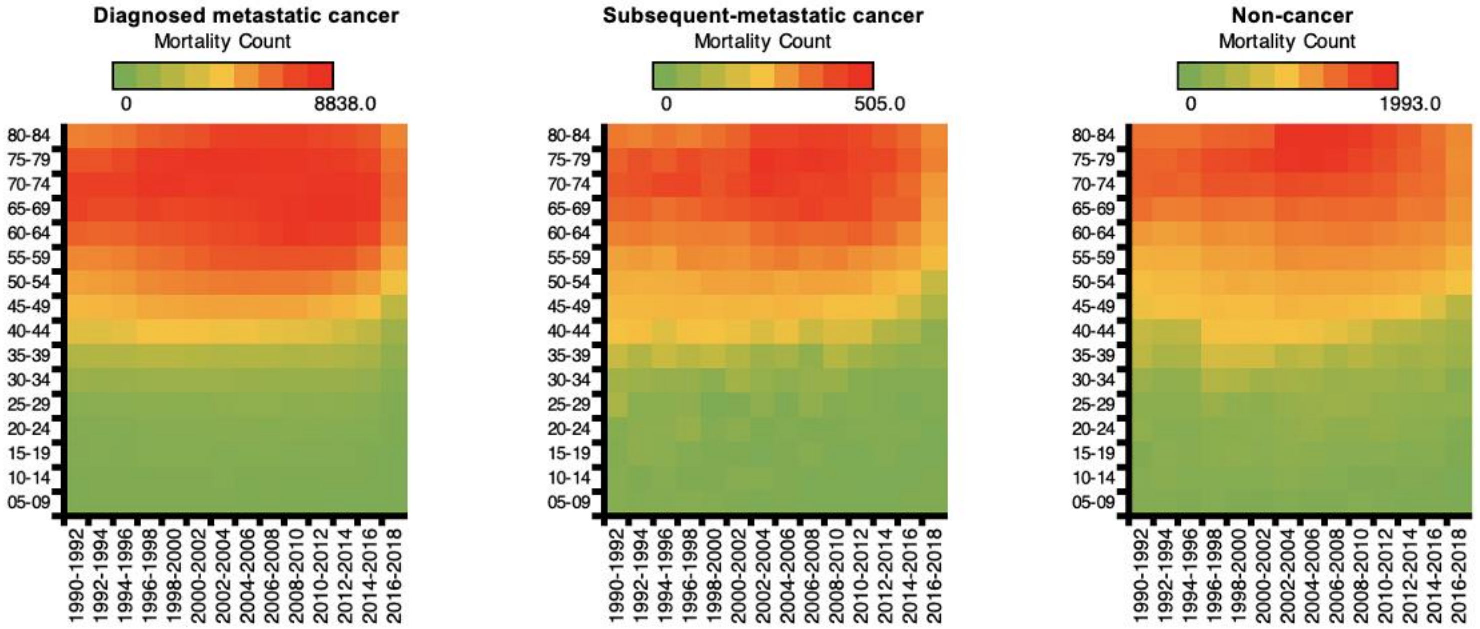

Supplemental Figure 4. Mortality counts attributed to diagnosed metastatic cancer, subsequent diagnosed cancer, and other causes of death as a function of age and year of diagnosis. The red, orange, and green colors represent the patients with highest, medium, and lowest risk due to mortality. The plurality of mortalities occur among patients >40 years old.

SUPPLEMENTARY FIGURE 5

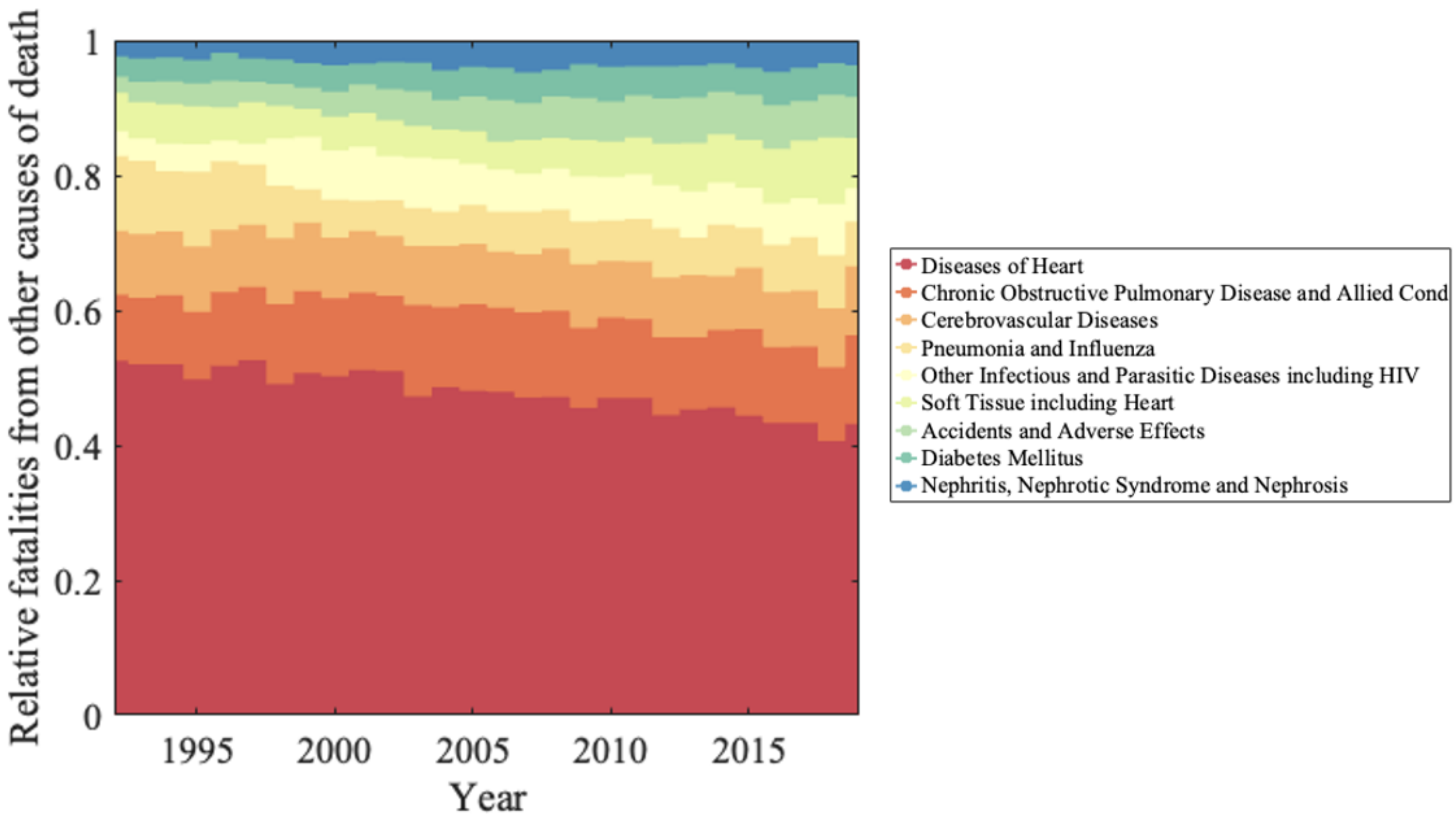

Supplemental Figure 5. Relative fatalities of the top ten contributing non-cancer causes of death among metastatic patients. The x-axis shows the year of diagnosis, and the y-axis shows the relative fatalities as a percentage.

SUPPLEMENTARY FIGURE 6

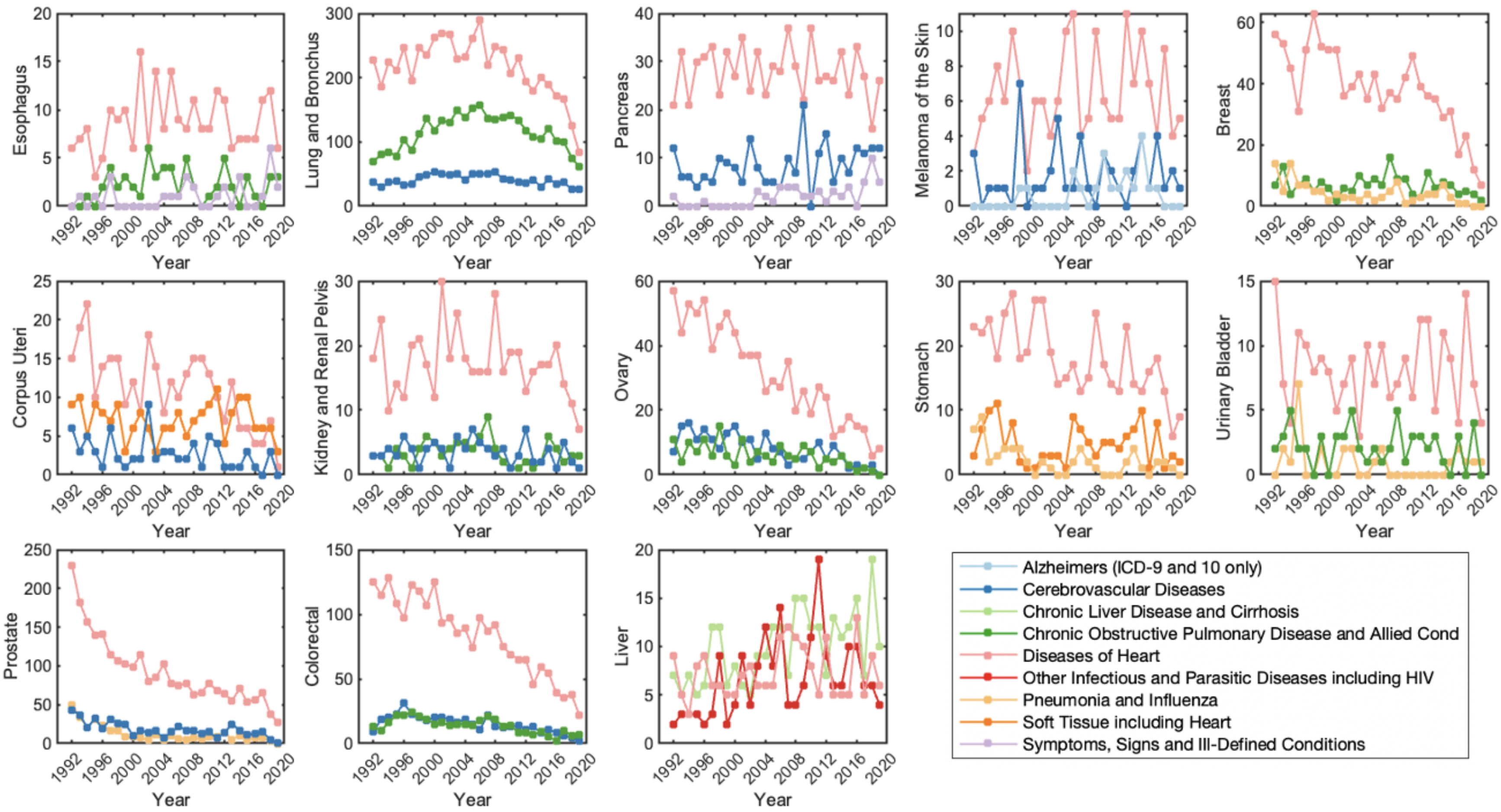

Supplemental Figure 6. Plots of top three non-cancer causes of death for various metastatic cancer subtypes (1992-2019). There are eight unique non-cancer causes of death that constitute the top three causes of death among the most prevalent metastatic subtypes: Alzheimer's (light blue), cerebrovascular diseases (dark blue), chronic liver diseases and cirrhosis (light green), COPD (dark green), diseases of the heart (pink), other infectious and parasitic diseases (red), pneumonia and influenza (yellow), soft tissue including heart (orange), and symptoms, signs and ill-defined conditions (violet).

SUPPLEMENTARY FIGURE 7

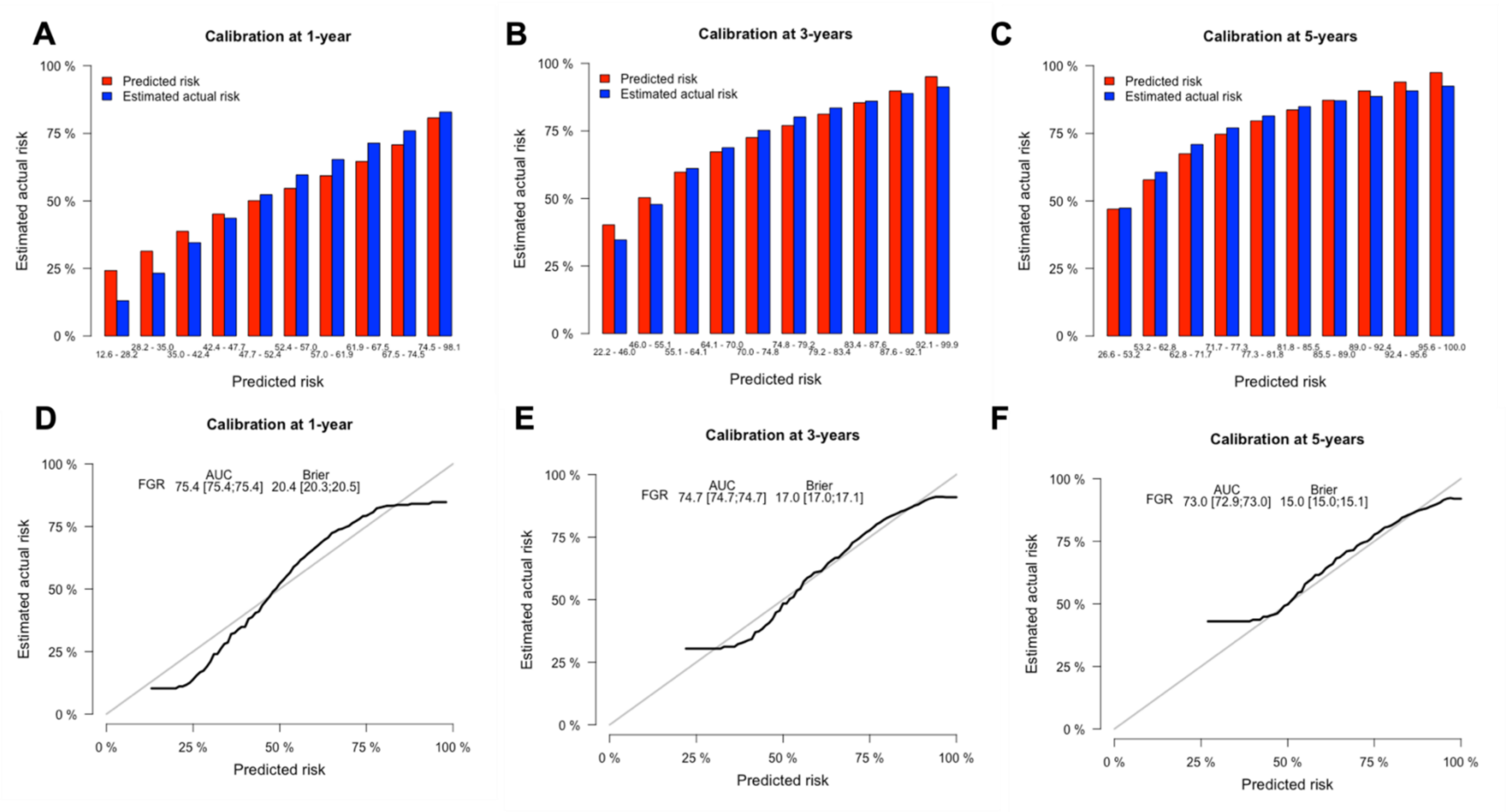

Supplemental Figure 7. Calibration plots and calculated Brier score (95% CI) of the selected Fine and Gray diagnosed cancer sub-distribution model. Data are adjusted for the competing risk of non-cancer death for: (**a**) 1-year risk (bar plot); (**b**) 3-year risk (bar plot); (**c**) 5-year risk (bar plot); (**d**) 1-year risk (line plot); (**e**) 3-year risk (line plot); and (**f**) 5-year risk (line plot).

SUPPLEMENTARY FIGURE 8

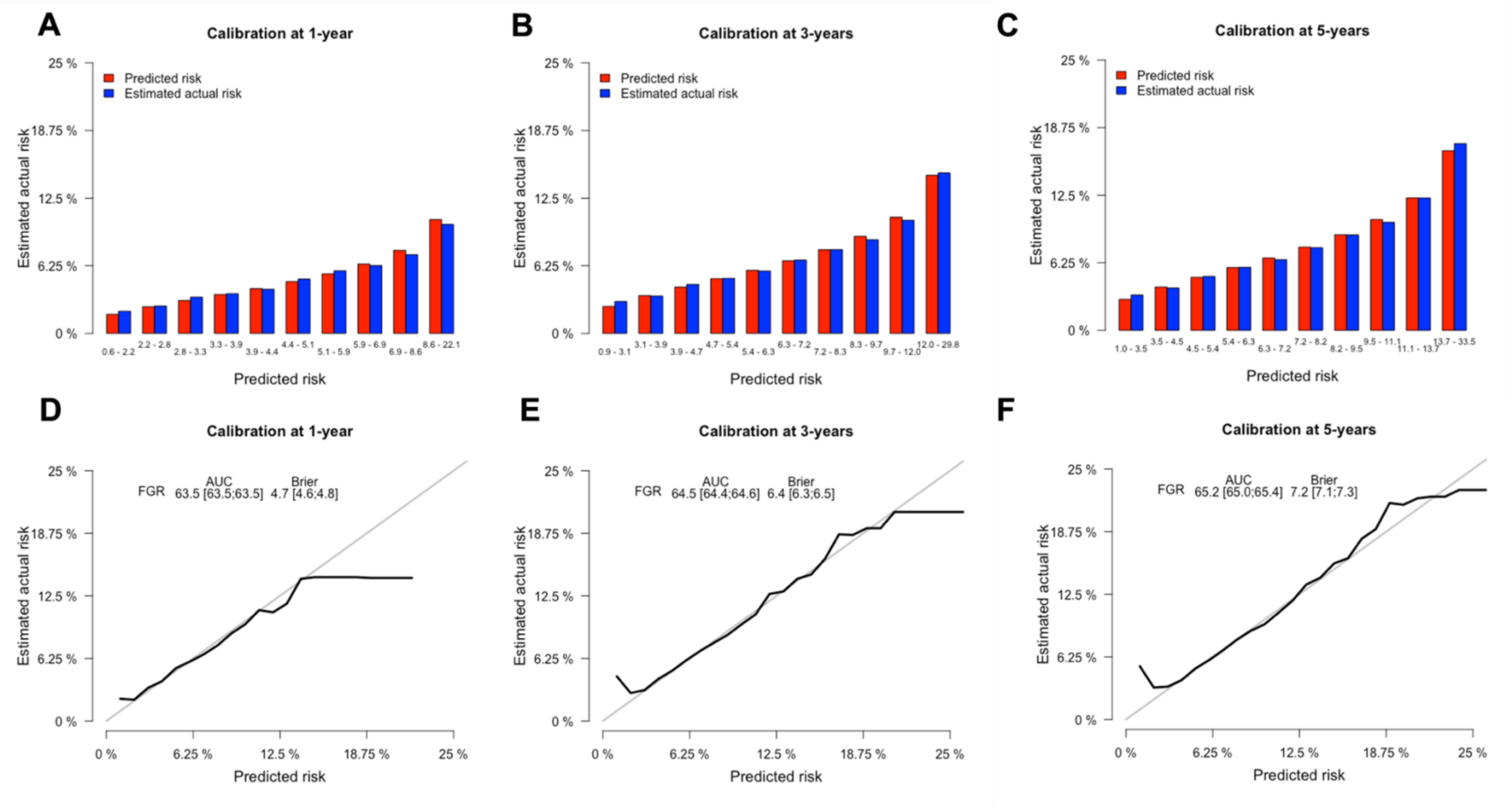

Supplemental Figure 8. Calibration plots and calculated Brier score (95% CI) of the selected Fine and Gray non-cancer sub-distribution model. Data are adjusted for the competing risk of diagnosed cancer death for: (**a**) 1-year risk (bar plot); (**b**) 3-year risk (bar plot); (**c**) 5-year risk (bar plot); (**d**) 1-year risk (line plot); (**e**) 3-year risk (line plot); and (**f**) 5-year risk (line plot).

SUPPLEMENTARY FIGURE 9

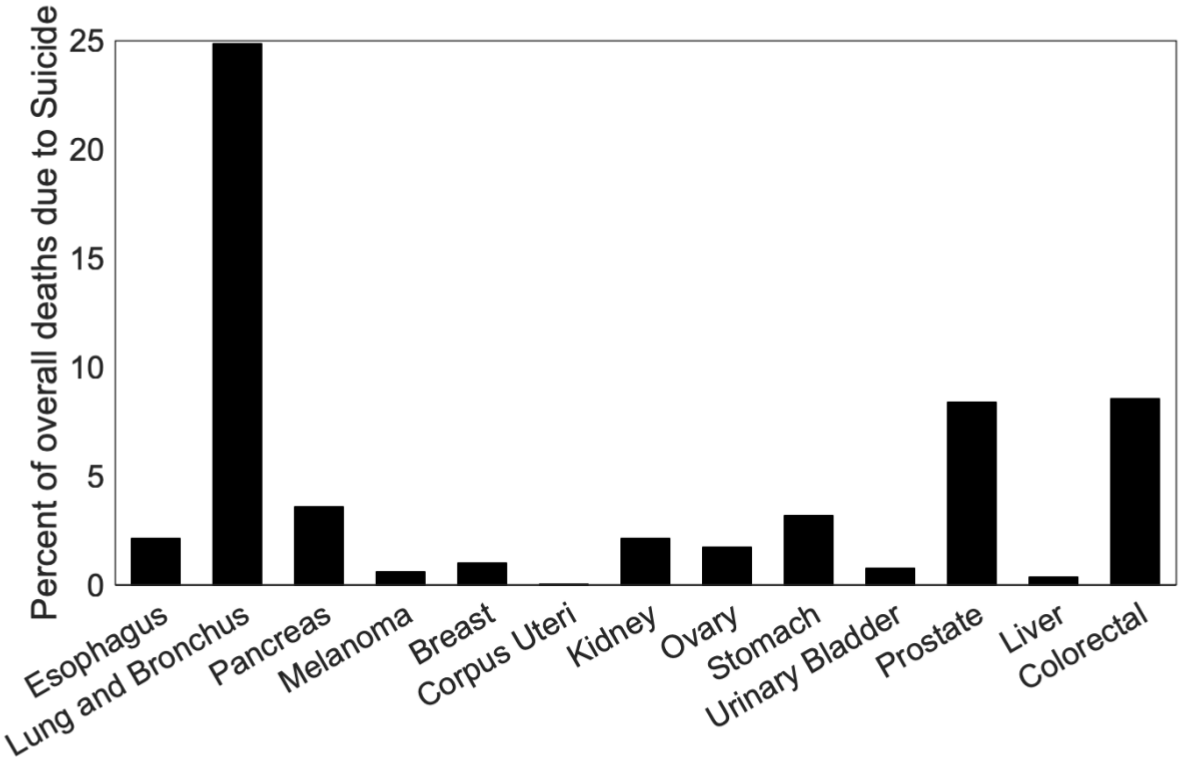

Supplemental Figure 9. Deaths due to suicide, stratified by the top 13 prevalent primary cancer-subtypes**.** The x-axis shows cancer subtype, while the y-axis shows the percentage of overall deaths due to suicide. Patients with metastases to the lung and bronchus, prostate, and colon and rectum constitute the plurality of patients who die due to suicide.

SUPPLEMENTARY REFERENCES

1 Park, H. S., Lloyd, S., Decker, R. H., Wilson, L. D. & Yu, J. B. Overview of the Surveillance, Epidemiology, and End Results database: evolution, data variables, and quality assurance. *Curr. Probl. Cancer* **36**, 183-190, doi:10.1016/j.currproblcancer.2012.03.007 (2012).

2 *National Cancer Institute. Casefinding Studies - SEER Quality Improvement.*, <http://seer.cancer.gov/qi/tools/casefinding.html> (2016).

3 Boer, R. *et al.* (Statistical Research and Applications Branch, NCI, Bethesda, MD).

4 Park, H. S., Lloyd, S., Decker, R. H., Wilson, L. D. & Yu, J. B. Limitations and biases of the Surveillance, Epidemiology, and End Results database. *Curr. Probl. Cancer* **36**, 216- 224, doi:10.1016/j.currproblcancer.2012.03.011 (2012).

5 Zaorsky, N. G. *et al.* Suicide among cancer patients. *Nature communications* **10**, 207, doi:10.1038/s41467-018-08170-1 (2019).

Zaorsky, N. G. *et al.* Causes of death among cancer patients. *Ann. Oncol.* **28**, 400-407, doi:10.1093/annonc/mdw604 (2017
