# Supplementary material for: Causes of death among people living with metastatic cancer
Source: Nat Commun. 2024 Feb 19;15:1519. doi: 10.1038/s41467-024-45307-x (PMC10876661; doi:10.1038/s41467-024-45307-x)
Supplement: Supplementary file 3 — Reporting Summary [file 41467_2024_45307_MOESM3_ESM.pdf]

nature portfolio

Corresponding author(s): Nicholas G. Zaorsky

Last updated by author(s): December 16, 2023

Reporting Summary

Nature Portfolio wishes to improve the reproducibility of the work that we publish. This form provides structure for consistency and transparency in reporting. For further information on Nature Portfolio policies, see our Editorial Policies and the Editorial Policy Checklist.

Statistics

For all statistical analyses, confirm that the following items are present in the figure legend, table legend, main text, or Methods section.

n/a	Confirmed	
☐	☒	The exact sample size (*n*) for each experimental group/condition, given as a discrete number and unit of measurement
☐	☒	A statement on whether measurements were taken from distinct samples or whether the same sample was measured repeatedly
☐	☒	The statistical test(s) used AND whether they are one- or two-sided<br>*Only common tests should be described solely by name; describe more complex techniques in the Methods section.*
☐	☒	A description of all covariates tested
☐	☒	A description of any assumptions or corrections, such as tests of normality and adjustment for multiple comparisons
☐	☒	A full description of the statistical parameters including central tendency (e.g. means) or other basic estimates (e.g. regression coefficient) AND variation (e.g. standard deviation) or associated estimates of uncertainty (e.g. confidence intervals)
☐	☒	For null hypothesis testing, the test statistic (e.g. *F*, *t*, *r*) with confidence intervals, effect sizes, degrees of freedom and *P* value noted<br>*Give P values as exact values whenever suitable.*
☒	☐	For Bayesian analysis, information on the choice of priors and Markov chain Monte Carlo settings
☒	☐	For hierarchical and complex designs, identification of the appropriate level for tests and full reporting of outcomes
☒	☐	Estimates of effect sizes (e.g. Cohen's *d*, Pearson's *r*), indicating how they were calculated

Our web collection on statistics for biologists contains articles on many of the points above.

Software and code

Policy information about availability of computer code

Data collection	The present study utilizes a three-part analytical strategy, as detailed in Supplemental Figure 1. Patient data for those diagnosed with metastatic cancer between 1992 and 2019 were extracted from the National Cancer Institute's Surveillance, Epidemiology, and End Results (SEER) program (SEER 18 database). The SEER program is a network of population-based incident tumor registries from various regions in the United States, representing 28% of the country's population, and includes information on incidence, survival, and treatment (such as radiation therapy, surgery, and chemotherapy). However, the SEER registry does not include information on comorbidities, performance status, surgical pathology, margin status, doses, or systemic agents. SEER*Stat 8.4.1 and and Microsoft Excel 16.0.1 (Microsoft, Redmond, WA) were used to collect the data in this study.
Data analysis	Data analysis was conducted using SEER*Stat 8.4.1, MATLAB R2022b (MathWorks, Inc., Natick, MA), and R Studio 3.3.0+ (R Studio Inc., Boston, MA). The 95% confidence intervals of SMRs were calculated using SEER*Stat 8.4.1.<br><br>CODE AVAILABILITY STATEMENT: The Fine and Gray survival calculator generated in this study can be accessed for external prediction and have been deposited under accession code: http://tinyurl.com/met-mortality. All other code used to perform statistical analyses or to train and validate the Fine and Gray models can be accessed at the following publicly available GitHub repository at ZENODO: https://doi.org/10.5281/zenodo.8422271.59

For manuscripts utilizing custom algorithms or software that are central to the research but not yet described in published literature, software must be made available to editors and reviewers. We strongly encourage code deposition in a community repository (e.g. GitHub). See the Nature Portfolio guidelines for submitting code & software for further information.

Data

Policy information about availability of data

All manuscripts must include a data availability statement. This statement should provide the following information, where applicable:

- Accession codes, unique identifiers, or web links for publicly available datasets
- A description of any restrictions on data availability
- For clinical datasets or third party data, please ensure that the statement adheres to our policy

DATA AVAILABILITY STATEMENT:

We comply with all relevant ethical regulations. The relevant session information used to abstract data and the user-submitted request and abbreviated data set (from SEER) for SMRs are provided in the Supplemental File. The individual patient-level SEER data are protected and are not available due to data privacy laws. However, the SEER patient data is publicly available under restricted access; access can be obtained via application form in compliance with relevant National Cancer Institute research use data agreements in the following repository: (https://seer.cancer.gov/data/access.html). These data are freely available and thus the study was exempt from institutional review board review. There are no participants in the study, and thus no consent form. The remaining data are available within the article and Supplementary Information file.

Research involving human participants, their data, or biological material

Policy information about studies with human participants or human data. See also policy information about sex, gender (identity/presentation), and sexual orientation and race, ethnicity and racism.

Reporting on sex and gender	We report sex as Male or Female. Further information on patient's gender (shaped by social and cultural circumstances) was not available in the SEER database at the time of data analysis. The sex variable was reported as collected directly from the SEER database. We performed appropriate sex-based analysis by accounting for patient Sex while stratifying for (1) death due to diagnosed cancer, (2) death due to non-cancer cause, (3) death due to subsequent cancer, and (4) death due to unknown cause.
Reporting on race, ethnicity, or other socially relevant groupings	Patients were appropriately stratified by Race including the following groups: White, Black, Asian or Pacific Islander, and American Indian/Alaska Native, as reported in the SEER database and in-line with the National Center of Health Statistics. This variable was not used as a proxy for socioeconomic status or any socially constructed variables.
Population characteristics	See below in Behavioral & social sciences study design section.
Recruitment	This was a large, population-based, cohort study that used national cancer registry data, and thus, recruitment was not applicable.
Ethics oversight	We comply with all relevant ethical regulations. The datasets generated and analyzed during the current study are available in the SEER repository (https://seer.cancer.gov/seerstat/). These data are freely available via the National Cancer Institute SEER program, and thus the study was exempt from institutional review board review. As the study did not include participants, there was no need for institutional review board review or consent forms.

Note that full information on the approval of the study protocol must also be provided in the manuscript.

Field-specific reporting

Please select the one below that is the best fit for your research. If you are not sure, read the appropriate sections before making your selection.

☐ Life sciences ☒ Behavioural & social sciences ☐ Ecological, evolutionary & environmental sciences

For a reference copy of the document with all sections, see nature.com/documents/nr-reporting-summary-flat.pdf

Behavioural & social sciences study design

All studies must disclose on these points even when the disclosure is negative.

Study description	The purposes of this work are to (I) characterize the causes of death among patients living with metastatic cancer as a function of disease site, year of diagnosis, and time after diagnosis and (II) predict the risk of death due to diagnosed metastatic cancer versus other causes of death (e.g., stroke, heart disease, etc.) at 1-, 3-, and 5-years after diagnosis. The study is a cross-sectional analysis involving only quantitative data. Patients with invasive metastatic disease, as coded in the SEER database, were included in the analyses. We looked at patients who died of 26 non-cancer causes of death and 15 primary cancers sites.
Research sample	Patient data for those diagnosed with metastatic cancer between 1992 and 2019 were extracted from the National Cancer Institute's Surveillance, Epidemiology. and End Results (SEER) program (SEER 18 database). Patients represented ages 0-85+, and the cohort was 49.2% female and 50.8% male. The patient cohort represented four races: White, Black, Asian/Pacific Islander, and American Indian/Alaskan Native. The methods and limits for each component of the analysis are outlined in the Supplemental Methods. To evaluate the impact of COVID-19 on mortality patterns, a detailed analysis of mortality counts of the 26 non-cancer causes of death

among patients with active follow-up in 2020 is presented in Supplemental Table 1. The SEER program is a network of population-based incident tumor registries from various regions in the USA, representing 28% of the country's population, and includes information on incidence, survival, and treatment. However, the SEER registry does not include information no comorbidities, performance status, surgical pathology, margin status, doses, or systemic agents.

Sampling strategy	All patients coded as having metastatic disease in the SEER database were included in the analysis (a total of 1,030,937 patients across 1992-2019). Of these, 82.6% of patients (n = 622,529) died due to the diagnosed cancer, while 17.4% (n = 145,006) died of competing causes. These sample sizes were not specifically chosen, but rather included all available data in the SEER database at the time that the data was originally abstracted.
Data collection	A case listing with metastatic disease, diagnosed from 1992 to 2019, was abstracted from the SEER program. Variables pulled included patient ID, age at diagnosis, year of diagnosis, race, sex, primary cancer site, presence of metastatic disease to the bone, liver, brain, or lung, survival months, cause of death, and vital status.
Timing	Data for this study were obtained from the SEER database from April-June of 2022; this includes the population-cohort presented in the main text. Additional data were pulled for subsequent analyses in June of 2023, and include the population-cohort included in the Supplemental Information (which includes data in 2020, as well).
Data exclusions	Patients diagnosed with cancer only through autopsy OR death certification were excluded (<1.5% of patients). Data were extracted for cancers with more than 10,000 person-years or more of survival time; thus, certain uncommon cancers were excluded, including male breast cancers, Mesotheliomas, and Kaposi's sarcoma. The cut-off point of 10-000 person-years of survival was established a priori. We selected the top 15 prevalent cancers and all 26 non-cancer causes of death in this analysis.
Non-participation	This is not a prospective study, but instead a retrospective, population-based, cohort study, and thus, non-participation is not applicable.
Randomization	This was a large population-based cohort study using all available patients from a national cancer registry, and thus, participants were not allocated to random groups. Further information on the data collection procedure can be found here: https://seer.cancer.gov/data/.

Reporting for specific materials, systems and methods

We require information from authors about some types of materials, experimental systems and methods used in many studies. Here, indicate whether each material, system or method listed is relevant to your study. If you are not sure if a list item applies to your research, read the appropriate section before selecting a response.

Materials & experimental systems

n/a	Involved in the study	
☒	☐	Antibodies
☒	☐	Eukaryotic cell lines
☒	☐	Palaeontology and archaeology
☒	☐	Animals and other organisms
☒	☐	Clinical data
☒	☐	Dual use research of concern
☒	☐	Plants

Methods

n/a	Involved in the study	
☒	☐	ChIP-seq
☒	☐	Flow cytometry
☒	☐	MRI-based neuroimaging
